# The Financial Costs of Mass Media Interventions Used for Improving Breastfeeding Practices in Bangladesh, Burkina Faso, Nigeria, and Vietnam

**DOI:** 10.3390/ijerph192416923

**Published:** 2022-12-16

**Authors:** Tina G. Sanghvi, Rick Homan, Thomas Forissier, Patricia Preware, Auwalu Kawu, Tuan T. Nguyen, Roger Mathisen

**Affiliations:** 1 Alive & Thrive Initiative, FHI Solutions, Washington, DC 20009, USA; 2 GHPR—Health Services Research, FHI 360, Durham, NC 27701, USA; 3 Alive & Thrive Initiative, FHI Solutions, New Delhi 110001, India; 4 Alive & Thrive Initiative, FHI Solutions, Abuja 900271, Nigeria; 5 Alive & Thrive East Asia Pacific, FHI Solutions/FHI 360, Hanoi 11022, Vietnam

**Keywords:** breastfeeding program, mass media, financial cost, Bangladesh, Burkina Faso, Nigeria, Vietnam

## Abstract

Breastfeeding is essential for child survival but globally less than fifty percent of infants receive adequate breastfeeding. Gaps in breastfeeding knowledge and misinformation are widespread. Mass media aims to motivate mothers and families, encourage care-seeking, improve social norms, and counteract misleading advertising. However, the costs and coverage of mass media are not well documented. Our study provides a cost-accounting of four large-scale mass media interventions and coverage obtained through mass media. We retrospectively calculated annual costs and costs per beneficiary of mass media interventions based on expenditure records in four countries. The interventions were a part of multi-component breastfeeding strategies in Bangladesh, Burkina Faso, Nigeria, and Vietnam. Annual costs ranged from 566,366 USD in Nigeria to 1,210,286 USD in Vietnam. The number of mothers of children under two years and pregnant women ranged from 685,257 to 5,566,882, and all designated recipients reached during the life of programs ranged from 1,439,040 to 11,690,453 in Burkina Faso and Bangladesh, respectively. The cost per mother varied from USD 0.13 USD in Bangladesh to 0.85 USD in Burkina Faso. Evaluations showed that mass media interventions reached high coverage and frequent exposure. This analysis documents the financial costs and budgetary needs for implementing mass media components of large-scale breastfeeding programs. It provides annual costs, cost structures, and coverage achieved through mass media interventions in four low- and middle-income countries.

## 1. Introduction

Evidence continues to grow that recommended breastfeeding practices have large impacts on newborn, infant, and child mortality, morbidity, growth, and cognitive development; maternal health; adult chronic diseases; and the environment [1,2,3]. Yet most of the world’s infants and young children are not adequately breastfed [4]. Factors that prevent breastfeeding from benefiting communities, mothers, and infants include widespread knowledge gaps in the population, weak social norms, women’s low self-efficacy, inadequate support from health providers and opinion leaders, lack of provisions for working women to breastfeed, and contradictory information used to market and promote alternatives to breastfeeding [5,6]. There is good evidence on what works to improve breastfeeding practices [5]. Mass media and social media were reported in a 2016 Lancet review on breastfeeding interventions as having a significant impact on the initiation of breastfeeding [5]. Mass media is one of the more frequently used channels for delivering health and nutrition education along with individual face-to-face counseling provided by health workers [5,7]. In recognition of the diverse influences that have an impact on breastfeeding, the 2016 Lancet review highlighted the need to motivate influential persons at the policy level and in the health system, reach persons in communities and families to shape feeding practices—in addition to reaching pregnant women and mothers—and recommended that large-scale programs should use mass media interventions and community mobilization [5]. However, not much is known about evidence-based large-scale mass media intervention design, costs, or coverage based on actual implementation. This gap has hampered progress in obtaining adequate resources and achieving large-scale coverage of breastfeeding programs. We address this issue in this paper. We analyzed mass media interventions that formed a part of multi-channel government-led programs to improve breastfeeding practices in Bangladesh, Burkina Faso, Nigeria, and Vietnam.

Although cost and coverage projections of mass media interventions for breastfeeding have been attempted in the past, the costs and coverage of mass media interventions based on actual in-country programs implemented at scale have not been previously reported [8]. This study strengthens the empirical basis of cost and coverage projections and for the first time provides a technical analysis of mass media interventions based on real program experience. Decision-makers have access to studies on the impacts and benefits of breastfeeding interventions, but they often question the high cost of mass media and, with no costing of actual large-scale mass media interventions available to guide them, they are unable to compare the investments needed to the well-documented losses from inadequate breastfeeding [9]. We based this analysis on expenditures incurred on mass media interventions that were implemented in four low- and middle-income countries (LMICs). The paper describes the mass media interventions, provides the costs and coverage achieved, and discusses factors influencing the total and per beneficiary costs across the four countries.

In Bangladesh, Burkina Faso, Nigeria, and Vietnam, mass media was one component of a larger social and behavior change (SBC) program and was accompanied by individual and group counseling provided by trained health workers and community volunteers to pregnant women and mothers of children below two years, and community mobilization (8). Face-to-face communication on breastfeeding was conducted by health workers in facility contacts and during home visits for antenatal care (ANC), postnatal care (PNC), and child health services. Social mobilization events were implemented for obtaining the support of community-influential persons such as family members, peers, village/town elders, and opinion leaders through individual and group meetings and video and theater shows. The dialogue was also conducted with national and regional government officials on policies for maternity leave and restrictions on harmful marketing of infant formula.

We refer to specific mass media-related actions as ‘interventions’ and the broader set of interventions as ‘programs.’ The term ‘intervention’ is used to indicate evidence-based actions designed intentionally to generate change and is based on the socio-ecological model of behavior change [10,11]. The term ‘mass media’ includes broadcast media (e.g., TV, film, radio, loudspeakers in vans), print materials used for public or mass consumption (e.g., billboards, posters, messages on buses), social media (e.g., Facebook, Twitter), and digital media (e.g., websites, digital displays in public). The mass media component was designed to reach mothers and key influential persons through channels selected based on media habits studies and varied across countries. The content and format of materials presented key audiences as role models and illustrated the type of desired support they should provide, in addition to conveying critical information on breastfeeding for mothers, family members, and other key audiences. Mass media was designed to produce independent and combined impacts by reinforcing other program interventions, such as motivating health workers to deliver better counseling, decision-makers to remove barriers through legislation, and reminding mothers to seek help for breastfeeding from health facilities.

In alignment with global evidence and national infant and young child feeding (IYCF) policies and strategies [5,12,13,14,15], the mass media breastfeeding interventions aimed to improve the early initiation of breastfeeding within the first hour of birth, six months of exclusive breastfeeding, and continued breastfeeding for at least two years [1,3]. Early initiation of breastfeeding required interventions to reach women during pregnancy and childbirth, while interventions for exclusive and continued breastfeeding needed to reach mothers of children below two years in addition to pregnant women. Specific categories of persons (e.g., health workers, family, and community members) who could enable or create barriers to breastfeeding were also targeted [5]. Breastfeeding promotion programs were based on behavioral science principles in all countries, adapted to country contexts through formative studies, and varied in the relative importance of various audience segments, content and how the content was conveyed, the number and types of channels, and the duration of exposure. The content of mass media addressed behavioral determinants (knowledge of how to practice each behavior, belief in benefits) and psychosocial factors (self-efficacy, perception of social norms, and family support) based on formative research studies and the application of social and behavior change principles. The implementation of mass media activities was largely subcontracted to private sector commercial market research, creative design, and broadcasting companies. An initial creative brief was prepared in collaboration with government authorities and other stakeholders to synthesize the findings of quantitative and qualitative formative studies. Decisions about the use of specific forms of mass media were based on media habit studies of mothers and influential persons. Channels used to broadcast the content ranged from one predominant channel (e.g., radio in Burkina Faso) to a broad mix in Vietnam consisting of radio, TV, loudspeakers in vans, social media, community theater, websites, and publicly displayed print materials (e.g., posters, messages on buses and billboards) [13,16]. Radio was also the most frequently used media in Nigeria with some TV, print, and social media. The total duration of program activities, including planning, research, strategy, media and materials development, and delivery and monitoring of interventions, varied from 43 months in Nigeria to 56 months in Bangladesh. The duration of exposure to mass media ranged from 19 months in Nigeria to 46 months in Bangladesh. The preparatory period of programs included conducting studies and assessments for strategic design and pre-testing; it was extended in several countries due to approvals and agreements and would be shorter if mass media interventions were conducted by government authorities.

Content and implementation strategies were adjusted during implementation if indicated by audience feedback. Most programs used data from a combination of embedded self-monitoring devices supplied routinely by media agencies and other mechanisms used by agencies to report media reach for different demographics. Special assessments were also conducted by locally hired teams. In Bangladesh, questions on media recall were added to other ongoing surveys to determine coverage and comprehension in various parts of the country. In Burkina Faso, international epidemiologists designed and analyzed the monitoring and trained the volunteer monitors who were paid a stipend. In Nigeria, a monitoring agency followed radio and TV broadcasts and trained community volunteers to document the content and frequency of airings reaching the community. Country differences in program content, structure, and management arrangements are explained by variations in program needs, local capacity in managing mass media interventions, resource constraints, and the diverse socio-cultural and media contexts of Bangladesh, Burkina Faso, Nigeria, and Vietnam. Existing breastfeeding levels and various stages of behavior change in countries played a role in shaping mass media interventions. Nigeria covered eleven states with spillover into nearby areas, while Bangladesh, Burkina Faso, and Vietnam achieved national coverage. Burkina Faso’s radio broadcasts were conducted in six languages; in Nigeria, the interventions were delivered in five languages; in Bangladesh in three; and in Vietnam, the materials were produced in two languages.

## 2. Materials and Methods

To develop cost estimates for this cost-accounting study, we extracted data retrospectively from accounting records on expenditures incurred for mass media-related activities in the four countries (Table 1). Details of mass media interventions were documented, and cost data were extracted for mass media activities related to breastfeeding promotion from program records. Program preparation activities and how the interventions were delivered and managed varied across the countries. We calculated expenditures on one-time startup activities that included planning, research, and strategy development. The recurring annual costs included production, broadcasting, printing, and placement of materials; multiple cycles of materials design; and monitoring activities. Management and administrative costs are included in all activities. The results of the expenditure analysis provide illustrative cost figures for donors, implementers, and governments to plan budgets for mass media interventions as part of comprehensive breastfeeding strategies. The study is based on actual real-life expenditure data on the development and implementation of mass media interventions, compiled retrospectively from accounting records in each country. The methodology involved separating specific mass media-related activities and costs incurred for breastfeeding promotion from broader IYCF programs. Mass media reach was obtained through survey data based on recall of mothers and their exposure to media and materials used by the programs.

Below we describe the resources used in developing and implementing mass media interventions for breastfeeding in the four countries, followed by how we calculated the costs of different operational components and costs of startup and recurring activities (Table 1). We then explain our methodology for assessing the number of beneficiaries and how we calculated the cost per beneficiary. The term ‘beneficiary’ includes mothers of children below two years and pregnant women who comprise the primary audience for mass media because they need to be supported and motivated throughout this period to follow age-specific breastfeeding recommendations, starting from the first one hour after delivery to two years of the child’s age. Secondary audiences that influence mothers’ breastfeeding practices, such as doctors, health workers, volunteers, family members, and peers, were reached through the same channels as mothers and specially developed media disseminated through channels used by them based on media habits studies.

### 2.1. Estimating Resource Use

Activities involved in the preparation of mass media interventions include planning, research, strategy development, and creative design and testing of materials. Descriptions of each of these steps and the types of resources used in each step are shown in Table 2. During the preparation phase, inputs consisted of the time of experts in developing and conducting research, meeting costs, travel costs for research, time of data analysts, the time of social behavior change and communication experts, time of creative designers of communication materials, photographers and filmmakers, actors and rental costs of film sets where audiovisuals were produced, printing and paper and prototypes of materials for field testing, and the time of managers to sub-contract, administer, supervise and report on the activities. The delivery of mass media involved the production of video and audio clips; printing of materials; paying for broadcasting time and outdoor display spaces, e.g., billboards; data collection for monitoring through surveys (time of interviewers and travel); analysis and interpretation of monitoring data; re-designing, field testing and producing new and revised materials; and the time of managers to sub-contract, administer, supervise, and report on the activities.

The sources of information on activities and resources include sub-contractor and project reports, publications on formative research, situational analysis reports, media audits, and media habits studies. Information on creative development processes for mass and social media was extracted from scopes of work and reports of firms contracted for design and development. Market rates were paid for buying media space in radio, TV, billboards, bus wraps, print, and digital poster displays. Media placement and monitoring information was extracted from contracts with media and advertising companies, and reports from firms producing monitoring results.

### 2.2. Cost Metrics

#### 2.2.1. Calculation of Total Costs by Country

We documented the expenditures incurred based on payments made for mass media activities. Where receipts were unavailable, resources were valued at market rates. The costing approach is a retrospective top-down expenditure analysis from a program planning perspective. The methodology is an accounting of financial expenditures rather than an economic analysis of costs [40]. Financial costing assesses items that entail monetary outlays and payments that will be needed, while economic costing assesses both monetary payments and the value of resources that are already paid for but assumed to be diverted from other activities; the value of what is given up represents an opportunity cost [40]. In each country, the first step in costing the mass media interventions was the identification of all activities that entailed monetary outlays and payments. A description of mass media activities was developed. Activities were broadly grouped into the preparation of interventions and delivery of interventions. The second step was the collection of data on the cost of these activities. Most activities were identifiable as mass media only, e.g., media audits, media habits studies, and media placement; others were conducted for the broader program and not only mass media (e.g., formative research). For some shared program expenses that were not identifiable as mass media, we allocated a share to mass media, based on the extent to which they supported those activities. Most mass media activities were sub-contracted to private companies, such as the design and development of materials for placement in radio, TV, social and digital media channels; media purchases (e.g., advertising time slots in radio and TV programs); printing and placement of billboard and poster materials; and media monitoring. All private companies incurred management costs, and these are built into activity costs obtained from accounting records of payments made. We layered on additional costs for administering and managing the sub-contracts at 25% of sub-contract costs for each activity. The actual overhead was 15%, calculated for the time and travel of managers, administrative and technical personnel, office rent, office supplies, and office communications. This estimate of administrative and management costs may be an underestimate as mass media activities were managed by the same managers and office staff who also managed several other interventions in the broader program (described under the intervention description above). We present cost estimates that use 25% and provide a range of costs that incorporate 15% to 40% administrative and managerial costs for programs with more limited interventions.

Expenditures incurred for each program component were entered into spreadsheets. Costs were allocated to the year that payments for them were recorded. Expenditures were classified as either start-up for those conducted once in the life of the program and only during the initial development of the program, or as recurring costs for activities that were repeated during the life of the program. The start-up activities include planning, situational analysis, formative research, media habits studies, and strategy development. Recurring costs include production, media placement and dissemination, monitoring, and ongoing changes in interventions based on findings from monitoring. Using the total expenditures incurred on mass media interventions, we calculated the proportion of costs attributable to four operational activities (preparatory studies, media design, media production and dissemination, and media monitoring). The mass media expenditures were converted to US dollars (USD) based on the prevailing exchange rate between the host country’s local currency unit (LCU) and USD for each calendar year. These expenses were then converted into 2019 USD using the Consumer Price Index ratio of the year of expenditure to the CPI in 2019 CPI data from the World Bank [41].

#### 2.2.2. Calculation of Average Annual Costs by Country

For comparative purposes, since program durations varied by country, we calculated average annual costs for each country. All start-up and recurring costs were aggregated to calculate total costs. The total costs incurred over the life of each country project were divided by the number of project years to calculate the average annual cost per year. This is important given that we need to compare annual costs with the annual number of beneficiaries reached.

### 2.3. Number of Beneficiaries

The number of beneficiaries consists of the number of mothers with children below two years of age and pregnant women. This was estimated from the population size of the program areas, the expected live births (birth rate per 1000 population), and less infant mortality (rate per 1000 live births) based on data from the World Bank [41]. Since the programs were aimed at reaching mothers of children below two years of age and pregnant women, we used the size of two birth cohorts plus one birth cohort for estimating the number of pregnant women for the total annual number. We then multiplied this result by the percentage of women who correctly recalled messages in household surveys; the number of beneficiaries is not based on the total eligible population but is based on the actual proportion of mothers who recalled specific messages and exposure to program media and materials. Based on the evidence of their role in influencing breastfeeding practices, we added the number of persons in key categories including health workers who provide breastfeeding information and support (one per 10 pregnant women and mothers of children under two years) based on evidence, when exposed to mass media, of better services and maternal knowledge [12]. In addition, we included one family member or peer group member in the community per pregnant woman or mother who was reached through mass media, as they were a key influential category in determining breastfeeding practices [42]. Two estimates of beneficiary reach are provided, one for pregnant women and mothers and the other with the addition of influential persons.

### 2.4. Calculation of Cost per Beneficiary

We divided the total cost from Table 3 by the total project duration in Table 2 and multiplied that by 12 to get a total annual cost, and then divided this by the number of beneficiaries to get the annual cost per beneficiary. We compared the total costs, average total costs per year, and cost per beneficiary per year across countries and examined differences in channels, content, duration, and administration/management of mass media interventions. We also compared differences in total costs over the life of programs, the relative proportion of program component costs to total costs, and the number of beneficiaries across country programs.

### 2.5. Sensitivity Analysis

Sensitivity analyses were carried out to examine the effects of assumptions and parameter uncertainty on the total costs and cost per beneficiary by including three variations of management and administrative costs (15%, 25%, 40%) and two variations of the number of beneficiaries by including pregnant women and mothers of children under two years with and without the addition of persons who influence breastfeeding practices that were targeted by mass media interventions.

## 3. Results

### 3.1. Total Costs by Country

The total financial outlays for mass media interventions implemented to improve breastfeeding were USD 3,445,630 in Bangladesh, USD 2,171,682 in Burkina Faso, USD 2,029,370 in Nigeria, and USD 4,942,002 in Vietnam (Table 2). Financial costs for the preparation of interventions, including design and planning, averaged USD 863,640 and took 18 months; the delivery of interventions including production, dissemination, and monitoring cost USD 2,283,531 and lasted for 31 months on average across the four countries. As a proportion of total mass media intervention costs, preparation costs accounted for 27.5% and the delivery of interventions accounted for 72.5% of total costs, on average. Most of the preparation costs (23.1% of total costs) were due to creative design, field testing, and revisions of materials that were developed in different regions in the countries and diverse languages and aimed to address knowledge gaps plus psychosocial factors driving the behaviors of mothers and other audiences. Media production, dissemination, and airtime for broadcasting accounted for 68.4% of the total expenditures on average and were lower in radio than TV.

### 3.2. Average Annual Costs by Country

The total annualized costs of mass media interventions were USD 738,349 in Bangladesh, USD 579,115 in Burkina Faso, USD 566,336 in Nigeria, and USD 1,210,286 in Vietnam (Table 3). Program durations varied from 43 months in Nigeria to 56 months in Bangladesh, averaging 49 months. The average annualized costs for all countries were USD 770,736 per year. Variations across the countries reflect the varying use of more or less expensive channels and the number of channels needed to achieve intensity or saturation, the market prices of media placement including airtime, and personnel costs.

### 3.3. Number of Beneficiaries by Country

Table 3 shows the annual number of beneficiaries by country who were exposed to mass media interventions to improve breastfeeding practices. This includes mothers of children below two years, pregnant women, and the number of selected additional audiences who were reached to enable mothers to practice breastfeeding as recommended. The proportion of eligible mothers actually reached by the mass media interventions were 70% in Vietnam, 62% in Bangladesh, 36% in Nigeria, and 34% in Burkina Faso. The number of mothers and pregnant women reached over the duration of the programs who recalled mass media content according to project evaluation surveys were 5.56 million in Bangladesh, 0.68 million in Burkina Faso, 2.45 million in Nigeria, and 3.19 million in Vietnam. With the inclusion of persons influencing breastfeeding practices, the total reach of mass media interventions in designated audiences was 11.69 million in Bangladesh, 1.44 million in Burkina Faso, 5.15 million in Nigeria, and 6.70 million in Vietnam. Differences among the countries are due to population size, media habits, the proportion of pregnant women and mothers of children under two years who recalled the content of mass media, and the duration of the programs.

### 3.4. Cost per Beneficiary by Country

The average annual cost per mother across countries and the cost per beneficiary, including all designated beneficiaries, is shown in Table 3. For individual countries, the cost per beneficiary was USD 0.13 in Bangladesh, USD 0.85 in Burkina Faso, USD 0.23 in Nigeria, and USD 0.38 in Vietnam. Both the annual costs and the annual number of beneficiaries varied across countries. The programs with the highest total costs were Bangladesh and Vietnam, but they did not have the highest cost per beneficiary due to their large populations, and Vietnam due to the high proportion of women who consume mass media.

### 3.5. Sensitivity Analysis

Varying the overhead costs for administration and management reduced the total average cost of mass media interventions across the countries by 8% if 15% was applied instead of 25%, and increased the total average cost of mass media interventions across countries by 12% if 40% was applied instead of 25%. The ranges of costs per beneficiary for pregnant women and mothers alone would vary from USD 0.24 to USD 0.29 over a range of 15–40% overheads, and from USD 0.11 to USD 0.14 when all intended beneficiaries reached are included. The cost per beneficiary was more sensitive to changes in the number of beneficiaries, and eliminating the additional beneficiary categories from the total reached by the mass media interventions increased the cost per beneficiary by 2.2 times.

## 4. Discussion

We documented the total costs, number of beneficiaries, and cost per beneficiary of four large-scale mass media interventions in Bangladesh, Burkina Faso, Nigeria, and Vietnam using retrospective cost-accounting of expenditures and coverage evaluations. To the best of our knowledge, this is the first detailed comparative analysis of mass media intervention designs, resource use, costs, cost structures, and reach/coverage targeted to breastfeeding promotion in diverse LMICs based on actual large-scale programs. In Bangladesh, the program was implemented over 56 months and cost a total of USD 3.4 million; in Burkina Faso, the program duration was 45 months and cost USD 2.2 million; in Nigeria, the duration was 43 months and cost USD 2.0 million; and in Vietnam, the duration was 49 months and cost USD 4.7 million. The largest proportion of expenditures was incurred for media production, dissemination, and monitoring at 72% on average; creative design and development accounted for 24% of the total, and the balance was for planning, strategy development, and monitoring. The number and types of mass media channels and duration of broadcasting and dissemination differed and accounted for variations in program delivery costs across countries. Bangladesh and Vietnam spent the largest proportion of total costs on media production and dissemination at 88% and 70%, respectively; this may reflect the predominant use of TV as well as longer program durations as compared to other countries. Administrative and management costs included in the total costs were estimated to range from 15% to 40% to reflect different scenarios where mass media is conducted as a stand-alone component (40%), rather than fully integrated with shared costs allocated across advocacy, interpersonal communication (IPC), and community mobilization interventions (15%).

The number of intended recipients varied with the geographic scale of the program (national in Bangladesh, Burkina Faso, and Vietnam, and in eleven states in Nigeria), and reflect the media habits of the population, in particular women with children below two years and pregnant women. These could partly explain the notable differences in costs. Since mass media is intended to reach influential persons who have proven impacts on feeding decisions and can enable women to practice breastfeeding, we included health workers, family members, and peers in the community among beneficiaries. The number of pregnant women and mothers of children below two years ranged from 685,000 in Burkina Faso to 5.5 million in Bangladesh; this number was 2.2 times greater when designated categories of influential persons were added. The cost per pregnant woman and mother (child under two years), ranged from USD 0.13 in Bangladesh to USD 0.85 in Burkina Faso. With the addition of key influential persons reached through mass media, the cost per beneficiary (all categories) was USD 0.06 to USD 0.40.

Few data on the costs or coverage of mass media interventions for breastfeeding are available in the literature other than hypothetical estimates. This paper is a “cost-accounting” exercise to document the actual costs of mass media used for breastfeeding promotion in four countries. Holla-Bhar et al. [43] attempted to make global projections for financial investments needed for implementing the 2003 WHO and UNICEF Global Strategy for IYCF and used USD 5 per live birth as a basis for calculating the mass media component. The rationale for applying this number was not stated. They suggest trying to match promotional costs of marketing breastmilk substitutes since the aim is “to counter industry messages undermining breastfeeding” and they also reference a 1992 World Bank report where Horton et al. recommend USD 5 per live birth [44]. Our expenditure analysis showed that mass media interventions cost on average USD 0.26 per pregnant woman or mother. Carroll et al. [8] compare the cost analyses by Holla-Bhar et al. and a more recent World Bank analysis by Shekar et al. [45] and discuss the strengths, limitations, and gaps in the two studies that estimate financial needs for scaling up breastfeeding interventions. They note differences in the types of interventions included and those interventions were defined differently. They conclude that both these methodologies produce broad financial approximations for planning at a global level but are limited when it comes to guiding specific country-level costs. Our study provides country-specific results based on actual implementation and describes details of the interventions to aid country-level planning and budgeting.

Comparing our costs with other breastfeeding interventions and multi-sectoral nutrition programs may provide insights into the component costs and cost structures of programs. The financial cost of two IPC programs in sub-Saharan Africa, based on the provider’s perspective suggests substantial economies of scale and lower per-beneficiary costs of mass media compared to IPC interventions. Desmond et al. costed a package of four antenatal breastfeeding counseling visits, plus fourteen breastfeeding counseling visits from birth to six months post-delivery in South Africa [46]. The incremental financial costs of a state-wide model (240,000 births per year) for two scenarios, including the optimal number of visits (clinic and home visits) and reduced number of visits (no home visits), were estimated retrospectively and ranged from USD 2 million to USD 7 million; this equals USD 8.3 to USD 29.3 per mother counseled. The authors found that over 90% of costs were for salaries of service providers, counselors, and managers at the provincial level; the omission of costs incurred by households to make clinic visits was noted as a limitation. Chola et al. estimated the cost of a community-based peer counseling district model (24,500 births per year) in Uganda where mothers were provided five visits by a peer counselor, starting in the seventh month of pregnancy through ten weeks post-delivery. The annual intervention costs of a more intensive and less intensive model ranged from USD 56,308 to USD 30,365 and the cost per mother was USD 139 to USD 74 [47] with an annual cost of USD 1.8 million for the scaled-up less intensive model (27). Personnel costs, particularly for supervisors of peer counselors, formed the largest share of intervention costs. In a prospective cost analysis conducted of two multi-sectoral stunting reduction programs in Guatemala and Burundi [48], the Behavior Change Communication (BCC) component was estimated to cost USD 4.7 million in Guatemala and USD 3.1 million in Burundi, as compared with our mass media costs that averaged USD 3.1 million. Monitoring costs in the multi-sectoral programs were also consistent with ours at 10.8% and 4.9% for Guatemala and Burundi, respectively, as compared to our monitoring costs that averaged 4.1%. However, the interventions and costs are not comparable, as the programs in Guatemala and Burundi were implemented in limited areas, involved multiple sectors and food distribution, and included different BCC components, while the mass media interventions in our analysis consist of a single category of interventions and reached a larger scale.

The analysis of costs in our study of four countries documents actual costs per beneficiary (Table 3) and may provide a rough benchmark for LMICs interested in getting a sense of similar programs in their countries, based on the media habits of their intended beneficiaries. In addition to aiding budget planning, our analysis of costs may contribute to preparing investment cases and estimating ‘global price tags’ [49]. For example, a recent model estimated that at existing inadequate levels of breastfeeding, Bangladesh loses USD 394 million annually, Burkina Faso loses USD 89 million, Nigeria loses USD 12,455 million and Vietnam loses USD 435 million annually. Scaling up breastfeeding could prevent illnesses and an estimated 800,000 deaths of children under five, 20,000 breast cancer deaths among mothers each year, and reduce hypertension and diabetes in women [1,2]. The cost of strengthening national efforts to improve breastfeeding through mass media interventions would be lower.

The use of mass media for improving public health practices is not new [50,51], although its application for breastfeeding promotion has not been reported comprehensively. The impact of mass media has been documented previously on child survival, smoking cessation, use of seatbelts, and other programs. Recently we have witnessed how mass media has been used to encourage vaccinations, social distancing, mask-wearing, and hand washing to reduce the transmission of the COVID-19 virus [52,53]. Based on a review of the global literature, the 2016 Lancet [5] recommended the inclusion of mass media in breastfeeding strategies to address ‘multifactorial determinants’ of breastfeeding. In most countries, mass media is used to achieve scale, usually covering populations of several million persons, and to reach not only mothers but also a broad cross-section of other people who influence the primary beneficiary. We documented evidence indicating potential for behavior change from mass media interventions in Bangladesh [12,42], Nigeria [15], and Vietnam [16]. Extracting the impact of mass media alone in a consistent and comparative way from the synergistic package of interventions has been a challenge. In Vietnam, women who reported exposure to the campaign were more likely than their unexposed counterparts to have breastfed exclusively for up to six months, and the difference between exposed and unexposed ranges from nine percentage points to eighteen at different times in the first six months. Exposure to mass media was associated with mothers’ beliefs that exclusive breastfeeding for 6 months was the norm (68% if exposed to mass media vs 46% if not); they believed that other mothers were giving only breastmilk (66% of mothers who had seen the spots and 47% if they had not been exposed). In Nigeria, infants had increased odds of being exclusively breastfed at 6 weeks if their mothers heard breastfeeding radio spots (OR 4.2, *p* = 0.029), discussed breastfeeding with a private health provider (OR 2.3, *p* < 0.001), or received text or WhatsApp messages about breastfeeding (OR 1.7, *p* = 0.048). In Bangladesh and Vietnam, exposure to mass media improved the performance of frontline workers in service delivery by improving knowledge and motivation; this in turn positively influenced mothers’ service utilization and knowledge of infant feeding [12]. The mass media interventions were designed for behavior change and used formative research approaches, rigorous field testing, and behavioral science theories and frameworks to address underlying drivers of breastfeeding practices, including targeting influential persons to create an enabling family, community, and health service environment. Hornik et al. have explored alternatives to costly research for streamlining preparatory costs [54]. The intensity of mass media (comprising frequency of exposures from diverse sources and sustained duration of multiple exposures) is linked with knowledge, beliefs, self-efficacy, social norms, and practices among beneficiaries (34). Broadcasting and dissemination costs of mass media are substantial; however, rapid increases in the consumption of social media can reduce mass media costs. Public-private partnerships with telecom companies and messenger applications such as WhatsApp, Facebook Messenger, Viber, TikTok, and YouTube need to be explored to offset government costs where possible. The selection and combination of channels, programs, timing, and placement for key audience segments also need to be strategically designed to gain value for money.

The need to find efficient solutions to address the infant formula industry’s threats to breastfeeding is rising in priority. According to WHO/UNICEF, “Many women express the desire to breastfeed, but a sustained flow of strategic and persuasive marketing messages undermines their confidence. Women’s positive attitudes towards formula milk correlate with their marketing exposure, and the fears and doubts they express about breastfeeding often mirror the themes and messaging of marketing” [6]. Global authorities recommend countries (governments, health professionals and their associations, civil society, and many other actors) to end the unethical marketing of formula products and support women, families, and caregivers in their infant feeding practices. Mass media interventions aimed to fill the information gap by providing accurate messages to key audiences.

Limitations of the study include not considering societal or beneficiary costs. However, the main contribution of this paper is to support program budgeting for mass media, using a strategy that optimizes existing channels and media exposure that are already used and does not incur new beneficiary costs. It is possible that some variability in costs is due to programs having been developed in different periods of time and having different durations. We may have missed costs incurred during the initial stages of building collaborations, obtaining feedback, and assessments used to decide on mass media. Our archived accounting records used to collect cost data did not provide input level costs, but gross amounts spent on intervention components; for this reason, we included detailed descriptions of activities in this paper and recommend ingredients-based costing conducted prospectively in the future. Potential underestimation bias might be introduced by separating mass media only from a program where mass media was designed to be a component. Fortunately, mass media is distinct from other components and more clearly identifiable and separable from other components in measuring costs. We report different types of impact data available from the programs that were measured differently across countries, but this was not the aim of the article and is a limitation of the study. A potential limitation is the lack of shared program costs and/or administrative costs, and we did not omit them and instead allocated a proportion from actual billings submitted to donors to cover these costs (15%) and presented 25% and 40% as two alternative scenarios for administrative and management costs. To address potential limitations in estimating the number of beneficiaries, we based our projections on survey data gathered from women of reproductive age. The term ‘beneficiaries’ is used to mean recipients who were intended to receive mass media and includes both pregnant women and mothers of children under two years, plus additional influential categories. We recognize that primary beneficiaries are pregnant women for initiating breastfeeding within the first hour of pregnancy and mothers (for breastfeeding for up to two years). However, additional persons are used to illustrate the cost per person achieved through mass media as reaching the influential persons would require community mobilization intervention costs if they were not reached through mass media.

## 5. Conclusions

Scaling up breastfeeding programs to protect infants and mothers in a country requires thoughtful consideration of mass media as a potential component due to economies of scale, and its ability to change perceptions and social norms. However, the resource needs are substantial and must be budgeted realistically. It can be a periodically intensified, ongoing core component of national breastfeeding strategies. This retrospective costing study of four mass media interventions for improving breastfeeding fills a serious gap and furthers our understanding of what resources are needed, how many beneficiaries can be reached, and provides examples of how to leverage the current and growing use of media to improve breastfeeding. Details on the content and structure of mass media interventions in different LMICs are provided as examples. We found that at USD 0.13 to USD 0.85 per beneficiary per year, the cost to governments and donors is minor compared with the cost of not achieving recommended breastfeeding practices that have been recently quantified. Selecting new mass media and social media channels that efficiently reach a high proportion of women and key influential audiences and using national expertise to design and implement programs can make mass media more affordable. Further research is needed to identify the minimum duration and frequency of media-based exposures required to achieve results and explore the synergies with interpersonal communication (counseling), community mobilization events, and legislative interventions to protect, promote, and support breastfeeding.

## Figures and Tables

**Table 1:** Program Components and Timing of Mass Media Interventions in Four Countries.

	Bangladesh 2009–2014	Burkina Faso 2015–2017	Nigeria 2017–2021	Vietnam 2009–2014
Total duration of mass media-related activities	56 months	35 months	43 months	49 months
Beneficiary exposure to mass media	46 months	27 months	19 months	31 months
(a) Planning, research, and strategy				
Situational analysis and formative studies	- Qualitative research: Rasheed et al., 2010 [17], Haider et al., 2010 [18] - Media audit: Nielsen 2009 [19] - Surveys: Saha et al. IFPRI 2010 [20], DHS 2007 [21]	- Qualitative research: PAMAC 2015 [22], - Survey: DMI 2014 [23], DHS 2012 [24], Wuehler et al., 2011 [25], Munos et al. 2014 [26]	- Qualitative research: Reboot & Picture Impact 2017 [27], Schnefke et al. RTI 2017 [28] - Surveys: Flax et al., RTI 2018 [29]	- Qualitative research: A&T 2015 [14,30]; Surveys: A&T [31], Nguyen et al. [32]
Strategy	- Joint stakeholder strategy with UNICEF and A&T assistance; including a mass media component IPHN/MOH 2010 [33]. - Micro-targeted doctor’s campaign was added later	- Pre-existing national scale-up plan for IYCF adapted MOH 2014 [34] - Comprehensive media plans developed by A&T & Development Media International (DMI)	- Joint stakeholder strategy: national and 2 state strategies developed MOH 2018 [35], A&T 2018 [36] including mass media components	- A&T supplemented the national policy, plan, and strategy MOH [37] with a comprehensive mass media component and a policy advocacy component
(b) Creative design and pretesting of materials				
Content of materials	- Problem-solving for EBF and EIBF, the role of HWs, in TV/radio spots - Overcoming common barriers in drama story format - Role models of supportive family, and community members [38] - Doctors motivated to counsel mothers	- Dramas on EBF and EIBF with families, community, HWs - Call-ins after each show - New content is continuously developed	- TV and radio spots used short dramas on EBF and EIBF emphasizing family and HW support - Positive role models [39] - Live radio and TV programs with experts and community influencers	- Short dramas on eliminating water from infant feeding, with scientific evidence - Confidence building for mothers to produce more milk by breastfeeding frequently [38]
Number of broadcast materials	- 3 TV spots in long (60–90 s) and 3 in short (20 s) forms - One animated film ‘Meena’ - 3 radio spots in story format	- 18 radio spots (60 s) in radio drama format - 2-h audience call-ins after the airing of spots	- 2 TV spots 60–90 s (per state for Lagos and Kaduna) - 2 radio spots per state - TV and radio programs, e.g., talk shows	- 2 TV spots (30 s and 45 s) - TV spots online - Audio messages delivered over outdoor loudspeakers
Additional mass media	- Newspaper inserts for doctors; animated film in government TV programs; TV spots shown in media dark communities using mobile generators	- None	- Social media messages, print materials e.g., pamphlets, posters in health centers, billboards; bus and tricycle branding	- Out-of-home: bus wraps, billboards, posters. - Digital: IYCF website, online counseling portal, Facebook fan page, mobile app for young mothers - Print: booklets, leaflets.
Languages	Bangla, Sylheti, Chittagonian	French, and 5 local languages	Pidgin, Hausa, Yoruba, Igbo, and English	Vietnamese and English (for almost all materials)
Creative design and research companies	Dhansiri (national advertising agency), Quantum (market research agency)	DMI, international creative development and research company based in London	McCann advertising agency’s Nigerian subsidiary STB-McCann, and national research agencies	Ogilvy advertising agency and national media and market research agencies
(c) Mass media production, placement, and dissemination				
Placement	- Commercial breaks in most watched TV and radio shows - Print media in newspapers, health centers	- Evening broadcasts attended by a broad range of community members	- Commercial breaks in most watched TV and radio shows - Billboards in high traffic areas and outside health centers, posters in health centers - Social media influencer platforms	- Commercial breaks in most watched TV shows - Websites, including 24 popular parenting websites and online interactive mothers’ forums - LCD screens in hospitals, health centers, and supermarkets. - Posters at health facilities.
Number of media channels	4 national TV channels, multiple national and regional radio stations	18 private radio stations covering high media coverage zones	7 radio stations, 7 TV channels in 2 states; expanded to reach 9 additional states. 25 experts trained to link the mass media to social media	18 national and provincial TV channels
Pattern of airing broadcasts	Intermittent, 3-month continuous, 3 times per year; aired 6 to 12 times a day, 3 days a week; intensity varied based on monitoring data	Broadcast 10 times each day, continuous for 6 months with a 5-month break and two 8-month segments	Intermittent, 30-sec radio/TV airings on average 5 days/week, modified based on monitoring	14 intermittent bursts over 31 months, most of which were 4 to 6 weeks long.
(d) Monitoring				
Data sources and use of data	- Monitoring devices embedded in randomly selected TVs sets in sampled homes to measure coverage - Consumer surveys, aided recall using still photos and audio clips - Questions added to A&T program assessment surveys for media recall	- Listeners hired in radio station coverage areas to determine compliance with planned airings - Rapid assessments and call-in talk shows provided feedback to revise and re-focus content	- Broadcast agency data on aired broadcasts for adjusting channels and airing frequency - Media monitors hired to confirm airings adhered to planned airings	- Media agency data on airings, and exposures by different demographic groups - Mid-term assessments through household surveys to document coverage and comprehension using aided recall
Management of monitoring activities	Media agencies, Nielsen survey agency	DMI research teams with contracted ‘listeners’ in radio coverage zones	Media firms, survey teams hired to collect exposure and recall data	Media placement agencies, national market research, and nutrition research agencies

A&T = Alive & Thrive, BRAC = Bangladesh NGO, DMI = Development Media International, EBF = exclusive breastfeeding, EIBF= early initiation of breastfeeding, IPHN = Institute of Public Health Nutrition in Bangladesh, PAMAC = Burkina Faso formative research institute, RTI-Research Triangle Institute.

**Table 2:** Costs ^1^ and Durations of Program Components of Mass Media Interventions by Country (2019 USD).

	Preparation of Interventions			Delivery of Interventions			Total	
	Planning, Research & Strategy	Creative Design & Pretesting of Materials	Duration (Months)	Media Production & Placement, Dissemination	Monitoring	Duration (Months)	Cost	Duration (Months)
Bangladesh								
Cost	$70,180 (64,566; 78,602)	$246,584 (226,858; 276,174)	10	$3,018,224 (2,776,766; 3,380,411)	$110,641 (101,790; 123,918)	46	$3,445,630 (3,169,979; 3,859,105)	56
% Of total	2.0%	7.1%		87.6%	3.2%			
Burkina Faso								
Cost	$123,392 (113,521; 138,199)	$725,514 (667,473; 812,576)	18	$1,084,128 (997,398; 1,214,224)	$238,647 (219,555; 267,285)	27	$2,171,682 (1,997,947; 2,432,283)	45
% Of total	5.7%	33.4%		49.9%	11.0%			
Nigeria								
Cost	$193,297 (177,833; 216,493)	$735,697 (676,841; 823,980)	24	$1,048,608 (964,720; 1,174,441)	$51,768 (47,627; 57,980)	19	$2,029,370 (1,867,021; 2,272,895)	43
% Of total	9.5%	36.2%		51.7%	2.6%			
Vietnam								
Cost	$164,990 (151,791; 184,789)	$1,194,904 (1,099,312; 1,338,292)	18	$3,465,766 (3,188,505; 3,881,658)	$116,342 (107,035; 130,303)	31	$4,942,002 (4,546,642; 5,535,042)	49
% Of total	3.3%	24.2%		70.1%	2.3%			
Overall								
Median	$144,191 (132,656; 161,494)	$730,605 (672,157; 818,278)	18	$2,051,176 (1,887,082; 2,297,317)	$113,491 (104,412; 127,110)	29	$2,808,656 (2,583,963; 3,145,694)	47
Average	$137,965 (126,928; 154,521)	$725,675 (667,621; 812,756)	18	$2,154,182 (1,981,847; 2,412,683)	$129,349 (119,002; 144,871)	31	$3,146,171 (2,895,397; 3,524,831)	49
Average % of total	4.4%	23.1%		68.4%	4.1%		100%	

Note: ^1^ All costs include 25% overhead (range represents 15%; 40% overhead).

**Table 3:** Total and Annual Costs ^1^, Beneficiaries ^2^, and Cost Per Beneficiary by Country (2019 USD).

	Program in Overall			Annual Number of Beneficiaries Reached			per Beneficiary Program Costs	
	Total Costs	Duration (Years)	Annual Costs	Pregnant Women or Mothers of Children Aged < 2 Years	Influential Family Members, Peers, and Frontline Workers	All Beneficiaries	Pregnant Women or Mothers	All Beneficiaries
Bangladesh	$33,445,630 (3,169,979; 3,859,105)	4.7	$738,349 (679,281; 826,951)	5,566,882	6,123,570	11,690,453	$0.13 (0.12; 0.15)	$0.06 (0.06; 0.07)
Burkina Faso	$2,171,682 (1,997,947; 2,432,283)	3.8	$579,115 (532,786; 648,609)	685,257	753,783	1,439,040	$0.85 (0.78; 0.95)	$0.40 (0.37; 0.45)
Nigeria	$2,029,370 (1,867,021; 2,272,895)	3.6	$566,336 (521,029; 634,296)	2,450,611	2,695,673	5,146,284	$0.23 (0.21; 0.26)	$0.11 (0.10; 0.12)
Vietnam	$4,942,002 (4,546,642; 5.535,042)	4.1	$1,210,286 (1,113,463; 1,355,521)	3,189,492	3,508,441	6,697,934	$0.38 (0.35; 0.42)	$0.18 (0.17; 0.20)
Median	$2,808,656 (2,583,963; 3,145,694)	3.9	$717,104 (659,735; 803,156)	2,820,052	3,102,057	5,922,109	$0.25 (0.23; 0.28)	$0.12 (0.11; 0.14)
Average	$3,141,171 (2,895,397; 3,524,831)	4.1	$770,736 (709,077; 863,224)	2,973,061	3,270,367	6,243,428	$0.26 (0.24; 0.29)	$0.12 (0.11; 0.14)

Notes: ^1^ Base case for costs assumes 25% overhead (range is based upon 15%; 40% overhead). ^2^ Beneficiaries calculated from live births minus infant mortalities from the last two years plus pregnant women expected in the current year multiplied by the proportion of women with access to mass media as reported in evaluation studies that measured maternal recall: 62% in Bangladesh [20] and 70% in Vietnam [32], 36% Nigeria [28], and 34% in Burkina Faso [23]. Mass media interventions in Nigeria covered 11 states and in Bangladesh, Burkina Faso, and Vietnam national programs were conducted; all programs were targeted at mothers as well as key influential persons. Based on media habits and coverage surveys, we estimated that for every mother of a child < 2 or currently pregnant woman, one additional influential family member or member of a peer group was reached; for every 10 mothers of children < 2 or currently pregnant, one frontline health worker was reached.

## Data Availability

Not applicable.
